# The Oldest Giant

**Published:** 1912-10

**Authors:** 


THE OLDEST GIANT. M. U. Sateschi, *Riv. Italiana du Heur., etc.,* Vol 4, 1911, cites a case of typic acromegalic gigantism, including complete anatomic and physiologic sexual infantilism, but with full mentality and good general health at the age of 76, the greatest age attained by any known giant. By X-rays, it appears that there is no increase of capacity of the sella turcica.

